# High-throughput sequencing technologies in the detection of livestock pathogens, diagnosis, and zoonotic surveillance

**DOI:** 10.1016/j.csbj.2022.09.028

**Published:** 2022-09-26

**Authors:** Godagama Gamaarachchige Dinesh Suminda, Srishti Bhandari, Yoonkyung Won, Umesh Goutam, Krishna Kanth Pulicherla, Young-Ok Son, Mrinmoy Ghosh

**Affiliations:** a Interdisciplinary Graduate Program in Advanced Convergence Technology and Science, Jeju National University, Jeju-si 63243, Republic of Korea; b Department of Molecular Biology and Genetic Engineering, School of Bioengineering and Biosciences, Lovely Professional University, Punjab, India; c Section of Surgical Sciences and Epithelial Biology Center, Vanderbilt University Medical Center, Nashville, TN, USA; d Department of Science and Technology, Ministry of Science and Technology, Govt. of India, Technology Bhavan, New Delhi, India; e Department of Animal Biotechnology, Faculty of Biotechnology, College of Applied Life Sciences, Jeju National University, Jeju-si 63243, Republic of Korea; f Bio-Health Materials Core-Facility Center, Jeju National University, Jeju-si 63243, Republic of Korea; g Department of Biotechnology, School of Bio, Chemical and Processing Engineering (SBCE), Kalasalin-gam Academy of Research and Educational, Krishnankoil 626126, India

**Keywords:** Domestic animals, High-throughput sequencing, Next-generation sequencing, Infectious diseases, Zoonotic pathogens, HTS, High-throughput sequencing, NGS, next-generation sequencing, PCR, polymerase chain reaction, qRT-PCR, quantitative reverse transcription polymerase chain reaction, BLAST, basic local alignment search tool, WG-NGS, whole-genome-NGS, BRD, Bovine respiratory disease, RABV, rabies virus, PGI2, Proteus genomic island 2, APEC, avian pathogenic E. coli, TCR, T-cell receptor, MRSA, Methicillin-resistant Staphylococcus aureus, WGS, whole-genome sequencing, GHSA, Global Health Security Agenda, mNGS, metagenomic next-generation sequencing, COVID-19, coronavirus disease 2019, ACE2, angiotensin-converting enzyme II, ILTV, infectious laryngotracheitis virus

## Abstract

Increasing globalization, agricultural intensification, urbanization, and climatic changes have resulted in a significant recent increase in emerging infectious zoonotic diseases. Zoonotic diseases are becoming more common, so innovative, effective, and integrative research is required to better understand their transmission, ecological implications, and dynamics at wildlife-human interfaces. High-throughput sequencing (HTS) methodologies have enormous potential for unraveling these contingencies and improving our understanding, but they are only now beginning to be realized in livestock research. This study investigates the current state of use of sequencing technologies in the detection of livestock pathogens such as bovine, dogs (*Canis lupus familiaris*), sheep (*Ovis aries*), pigs (*Sus scrofa*), horses (*Equus caballus*), chicken (*Gallus gallus domesticus*), and ducks (*Anatidae*) as well as how it can improve the monitoring and detection of zoonotic infections. We also described several high-throughput sequencing approaches for improved detection of known, unknown, and emerging infectious agents, resulting in better infectious disease diagnosis, as well as surveillance of zoonotic infectious diseases. In the coming years, the continued advancement of sequencing technologies will improve livestock research and hasten the development of various new genomic and technological studies on farm animals.

## Graphical abstract

Overview of impact of sequencing technologies and its applications in livestock research.

**Figure:**
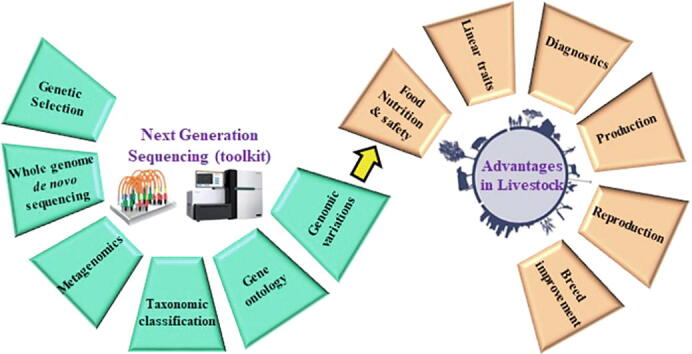

## 1 Introduction

The ongoing development of high-throughput sequencing (HTS; also known as next-generation sequencing) technologies has resulted in a dramatic reduction in DNA sequencing costs, making the technology more accessible to the average. The amount of DNA sequence data that can now be produced using next-generation sequencing (NGS) platforms (Ion Torrent/Proton/Personal Genome Machine sequencing, Roche-454 GS Junior/FLX+, and Illumina HISeq/MiSeq/GAIIx) is a clear example of this step-change. Similarly, recent advances in protein and peptide separation efficiencies, as well as highly accurate mass spectrometry, have facilitated protein identification and quantification in a given sample. These biotechnology advances are increasingly being applied to the study of animal infectious diseases, and they are beginning to revolutionize the way biological and evolutionary processes can be studied at the molecular level.

Emerging infectious diseases have significantly risen in recent years owing to rising globalization, intensifying agriculture, urbanization, and climatic changes. Over the last few decades, emerging infectious diseases, such as avian influenza, African swine fever, foot and mouth disease, and bovine spongiform encephalopathy, have been associated with domestic and companion animals, and have caused major losses to livestock rearing communities, in addition to posing significant threats to human and animal health [1], [2], [3], [4]. Therefore, zoonotic pathogens should be considered key components of the global health system. In recent decades, invasions from animal and livestock reservoirs have led to high-impact epidemics [5]. Specifically, livestock diseases cause economic losses through direct and indirect expenses, have substantial social and environmental impacts, and threaten global food security [6]. The direct cost of zoonotic illnesses has been estimated at more than $20 billion, while indirect losses to impacted economies amount to over $200 billion [7].

To address diverse diagnostic issues, such as the genetic relationships between bacteria or viruses related to livestock or companion animals, the detection of mutations in viral or bacterial genomes that lead to resistance against antivirals or antibiotics, numerous companies are developing relatively affordable, rapid, and smaller devices based on advanced technologies for the rapid detection of infections in domestic animals [8].

The second and third-generation sequencing platforms provide numerous benefits over traditional microbiological diagnostic techniques, including the ability to detect fastidious or non-culturable pathogens and co-infections. The HTS enables researchers to simultaneously identify a wide range of DNA sequences, either using a specific genetic region (metabarcoding/amplicon-based methods) or all genetic material. Moreover, sequencing technologies have been used to characterize viral diversity in humans and animals. From epidemiology to the evolution of viral quasispecies to metagenomic characterization of unknown pathogens or microbial communities, molecular identification has significant implications.

Since the field has rapidly developed owing to continuous improvement and refinement of existing systems as well as the release of completely new platforms, resulting in a dramatic reduction in DNA sequencing costs, making the technology more accessible to the average laboratory. In this article, we attempted to summarize the use of sequencing simulations (HTS) in veterinary research, i.e., an in-depth discussion on HTS-based technologies in the detection of livestock pathogens (Bovine, *Canis lupus* familiaris, *Ovis aries*, pigs, horses, chickens, and ducks). Further prospective aspects of HTS are also discussed, with a focus on improving monitoring and detection of zoonotic infections, as well as diagnosis and control of infectious zoonotic diseases.

## 2 Conventional assays in veterinary microbiology diagnostic

Traditional testing methods are still used in veterinary medicine [9] (Table 1). Traditional serology, cell culture, electron microscopy-based methods, virus neutralization, immunodiffusion, and immunoassay techniques for diagnosing animal diseases are either time-consuming or labor-intensive. Currently, immunological assay-based techniques as well as various molecular detection-based techniques allow for better diagnosis. In addition, the expression of pathogen-specific proteins has enabled the development of assays that can distinguish vaccinated from non-vaccinated animals.

**Table 1:** Diagnostic mythologies used in detection of infection agents in host animals.

Diagnostic tool kit/ technology	Name of diagnostic kit/ technology	Description
Specific Toolkits	Antigen rapid rabies Ag test	Detection of rabies virus using antibodies in dogs
	SNAP Parvo test	Detection of parvovirus in dogs
	SNAP BVDV kit	Detection of bovine diarrhea in cattle
	BioSign FMDV	Detection of Foot and Mouth disease in ruminants
Basic Diagnostic technology	PCR	Amplification of pathogen nucleic acid
	qPCR / RT-PCR	Diagnosis pathogen
	Sequencing	Detection of pathogenic by high-throughput sequencing

Biosensor-based assays involve a receptor for the target pathogen and a transducer to detect the signal for the disease. Gamma interferon assays are used to detect tuberculosis in primates, cattle, and cervids [10], [11], [12]. However, the high cost of instruments and sample analyses in these methods limit robust testing, hamper early detection, and curb the spread of disease. Besides, these techniques are not adaptable for use in the field with portable instruments and reagents.

In recent years, the exponential increase in the applications of nucleic acid-based diagnostic techniques has redefined the quality of information available for animal disease control programs. Transgenic plants are also used to express veterinary pathogen proteins and are predominantly used to produce large amounts of recombinant proteins [13], [14].

They are extensively used for genotyping and phylogenetic analysis of veterinary pathogens. Initially, polymerase chain reaction (PCR)-based methods were used for large-scale diagnosis and rapid identification of avian influenza-specific genomes. qRT-PCR (quantitative reverse transcription polymerase chain reaction) is a single-tube closed technique that can be used in veterinary medicine. Portable adaptations for use in the field may allow for rapid decision-making during an emergency outbreak.

The sequencing technology, which was initially developed for mapping genes, has been predominantly applied to develop or improve therapeutics to detect a wide variety of veterinary pathogens [8], [15]. The NGS techniques (both second generation and third generation sequencing methods), employ various methodologies (e.g. metagenomics, whole-genome sequencing, and transcriptomic analysis) that have led to improvements in the field of genetic research. NGS-based metagenomics and follow-up PCR-based tests targeting identified pathogen sequences are combined with more traditional diagnostic approaches such as isolation and characterization. This is critical in circumstances when metagenomic data suggest the existence of several diseases [16].

## 3 Overview of pathogen–host interactions and zoonosis infection

Zoonosis is a disease or infection that can be transmitted from animals to humans [17]. Zoonoses constitute a significant public health issue worldwide because of the close relationship of humans with animals. Approximately 60 % of infectious diseases have origins in zoonotic pathogens [18]. According to the Centers for Disease Control and Prevention, most zoonotic diseases are bacterial (41.4 %), followed by viral (37.7 %), parasitic (18.3 %), fungal (2 %), and prionic (0.8 %), based on surveillance data [19]. Zoonotic diseases account for approximately 2.5 billion human illness cases and 2.7 million deaths annually [20]. In addition, approximately 60,000 people die from rabies, avian influenza, Ebola, and Rift Valley fever annually [21], and there are approximately 200 known zoonoses [22]. Zoonotic influenza, salmonellosis, West Nile virus, plague, emerging coronaviruses, rabies, brucellosis, and Lyme disease are the major zoonotic diseases of concern in the United States [23]. Zoonoses comprise a large percentage of new and existing diseases in humans [18]. Some diseases, such as HIV, originated as zoonosis, but later mutated into human-only strains. Other zoonoses, such as Ebola, SARS-CoV-2 and salmonellosis, can cause recurring disease outbreaks [24], [25].

Depending on the mechanisms of transmission and epidemiology, zoonoses can be classified into the following four major types: cyclozoonoses (e.g., tapeworm infections), metazoonoses (e.g., arboviral and trypanosomal diseases), saprozoonoses (e.g., histoplasmosis), and direct zoonoses (e.g., plague, salmonellosis, leptospirosis, and rabies). Further, zoonotic diseases may be classified based on the route of transmission, pathogen type, and degree of contagious [26]. Microbial and virion particles influence zoonotic infection in animals and humans due to their influence and invasion and adaptation activities [27]. Bacterial zoonotic diseases can be transferred to humans by ecological changes in the human environment, animal handling, animal by-products, and infected animal wastes, such as saliva, blood, urine, and feces. Bacterial pathogen transmission is caused by ingestion or through bacterial invasion of the skin epidermis [28], [29].

The discovery of antibiotics has improved health management and our understanding of infectious diseases. However, caution should be exercised when using antibiotics in domestic animals [30]. Agricultural workers in areas with high use of antibiotics in farm animals may be at an increased risk of infection with antibiotic-resistant pathogens. Antimicrobial resistance is a complicated factor in the control and prevention of zoonoses. There is widespread use of antibiotics in animals reared for food, which increases the potential for the spread of drug-resistant strains of zoonotic pathogens in animal and human populations. A microbial-based genetic study revealed the presence of antimicrobial resistance gene *mcr-1* in three *E. coli* strains isolated from retail chicken meat (Table 2). The *mcr-1* gene is likely acquired by humans through the consumption of *mcr-1*-containing retail meat [31], [32], [33]. While antibiotic use in food animals may pose a risk to human health, sensitive and early diagnosis is one of the most critical components for effective response to infectious disease threats. WHO surveillance data predict that the number of deaths caused by resistance to antibiotics, including multidrug resistance, will outnumber the number of deaths from cancer in 2050 [34]. The safe and sustainable treatment of animals and humans has become a global challenge. Common pathogens and their antibiotic resistance genes are listed in Table 2.

**Table 2:** Common pathogens and their antibiotic resistance genes.

Disease	Pathogen	Route of infection	Antibiotic resistance genes	Transfer source	References
Bovine tuberculosis	*Mycobacterium bovis,* *Mycobacterium tuberculosis*	Drinking infected milk, inhaling the bacterium shed by infected animals, or direct bacterial contact with a cut or other breaks in the skin	*pncA, embB, katG, inhA, rpoB, rrs, gyrA, gyrB*	Respiratory secretions, feces, milk	[120], [121], [122], [123], [124]
Salmonellosis	*Salmonella* sp.	Gastroenteritis	*mcr-1* ESBL-CTX-M-1, ESBL-CTX-M-2, ESBL-CTX-M-9 ESBL-CTX-M-15 ESBL-CTX-M-65, ESBL-TEM-52, CMY-2, CipR	Pork products, poultry, seafood, milk, chicken, eggs	[120], [121]
Leptospirosis	*Leptospira interrogans*	Skin or mucous membranes (eyes, nose, or mouth), especially if the skin is broken from a cut or scratch	*rpsL,* *rrs*	Contaminated water, animal urine, contaminated food	[120], [125]
Foodborne disease	*Escherichia coli*	Contaminated water, contaminated food, oral-fecal route	ESBL, ESBL-CTX-M-1, ESBL-CTX-M-9, TEM-52, CMY-2-AmpC, aadA12, aadA13, aadA7	Livestock, water, person-to-person	[121]
	*Enterobacteriaceae*		bla_CTX_M_, bla_TEM,,_ bla_SHV,_ bla_OXA-10,_ bla_OXA-13,_ bla_CMY,_ bla_LAT,_ bla_ACC,_ bla_ACT,_ bla_MIR-1,_ bla_DHA_		[126], [127], [128], [129], [130]
Bumblefoot / foodborne disease	*Staphylococcus aureus*	skin and mucous	mecA, mecB, mecC, mecD	infected blood or body fluids	[131]
Pneumonia/ Foodborne disease	*Pseudomonas aeruginosa*	skin penetration, contaminated food	*ampC, mexX, mexL, mexR, nfxB, or mexZ*	soil, water, and humid environments	[132]
Brucellosis	*Brucella melitensis*	eating or drinking inhaling	*rpoB*	Contaminated water, contaminated food (milk, meat)	[133]

**Fig. 1:**
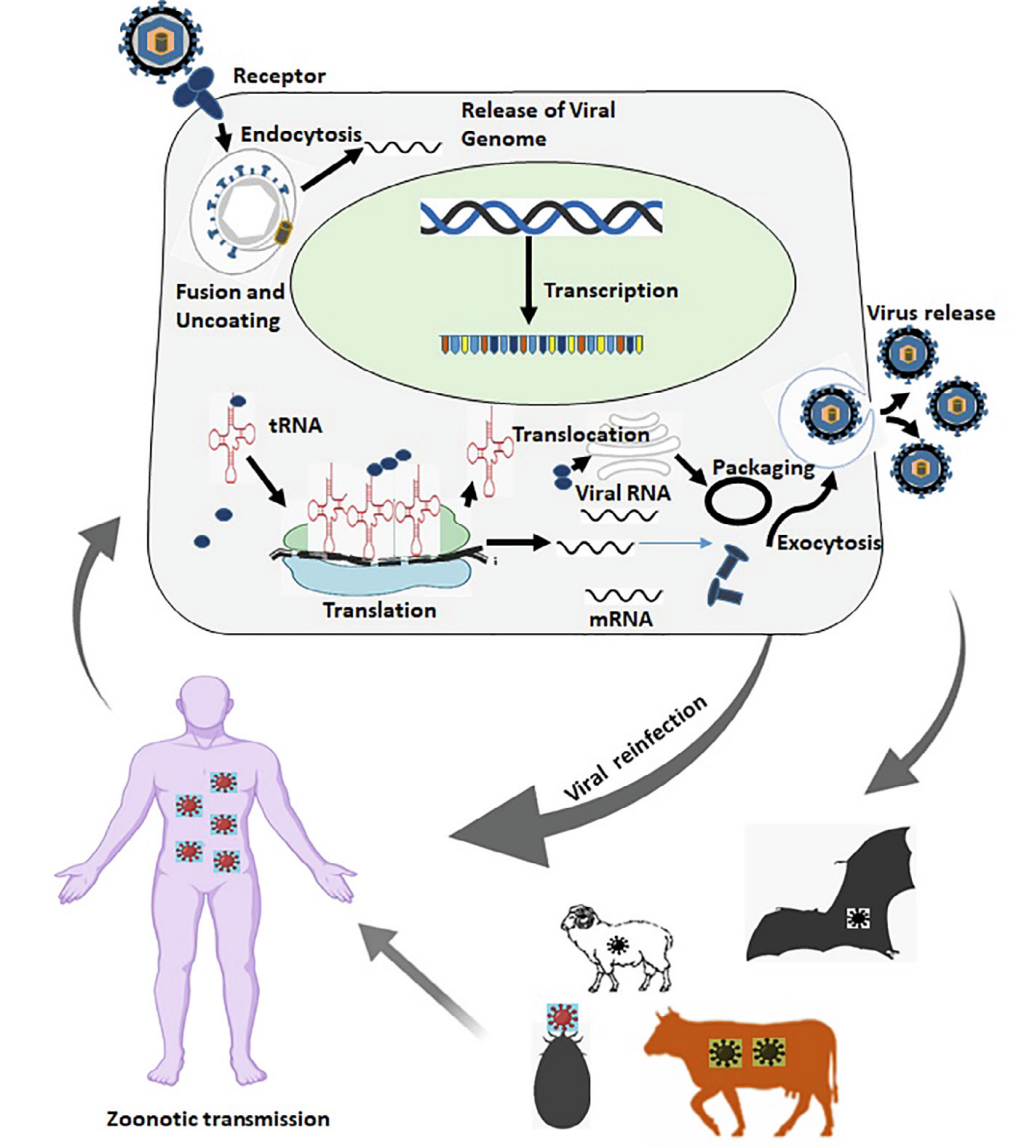
Prospective illustration of virus replication mechanisms and spread of zoonotic virus. Many zoonoses are presently under control, however there remain gaps in our understanding of many illnesses, including disease distribution, aetiology, pathogen, host, vector biology, dynamics, transmission cycle, risk factors, and predisposing factors.

Outbreaks of zoonotic infectious disease or reverse zoonotic disease transmission (zooanthroponosis) in humans are caused by the spillover (cross-species spillover) of pathogens from animals, and locations where individuals and animals frequently interact are potential spillover sites [35]. Pathogen potency, host immune defense, and human activities affect spillover events [36]. Several factors determine the success of viral infection, including host support for sufficient virion production, accessibility to enter the host, host receptors, and tropism [37]. The primary barrier to virus entry into the host body is the epithelium. The mucosal layer is another protective layer in the gastrointestinal tract. Virus entry points vary across different viruses, and some viruses can cross the placenta and infect the fetus. After entering the host’s body, viruses bind to the host cell receptors, escape the immune system, and proliferate inside host cells (Fig. 1) [37]. Associated with potential consequences of spillover events caused by rapid mutation and the conquering of sensitivity to existing treatment, sequencing approaches should be used to reduce the impact of such viruses on humans.

Overall, the outbreaks of highly pathogenic diseases have mainly originated in the livestock industry and some of them have caused huge losses [38]. The zoonotic is not only public health concerns but also has socioeconomic impacts based on their potential adverse effects on livestock productivity. For example, zoonotic diseases decrease the quality of animal products, such as milk, eggs, and meat, and may even cause death. Zoonoses also affect the global trade in animal products for food and other applications. Cows (*Bos taurus*), pigs (*Sus scrofa*), sheep (*Ovis aries*), goats (*Capra hircus*), chicken (*Gallus gallus* domesticus), and ducks (*Anatidae*) are associated with primary agricultural production.

## 4 Combination of bioinformatics simulations with HTS in veterinary research

**Fig. 2:**
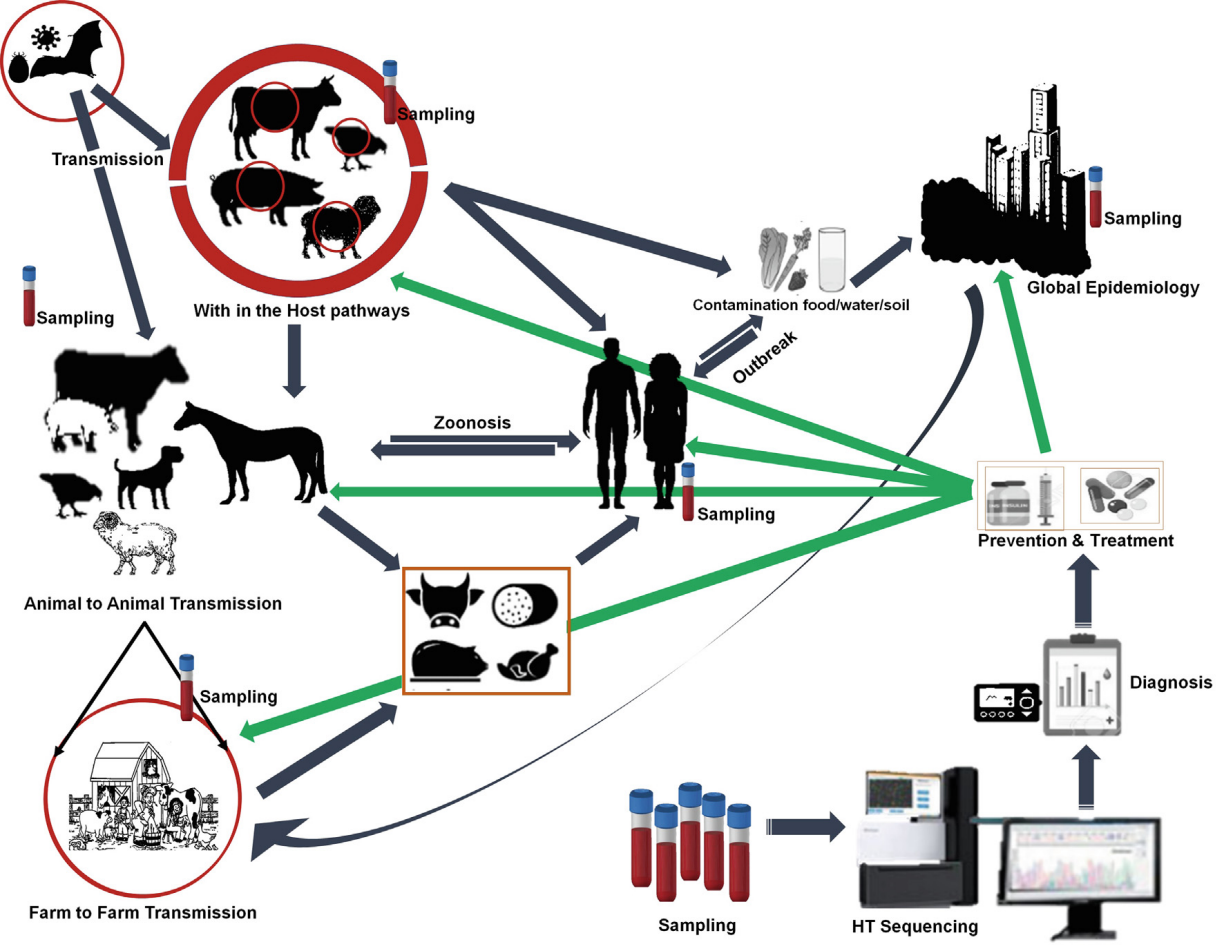
Schematic diagram summarizing the applications of HTS for screening, control and management of epidemic outbreak. The etiological agents may infect with in the host and in advanced phases, these pathogenic agents induce worldwide epidemiology via re-transmission and amplification in different livestock populations and emerging and re-emerging zoonoses. Priority is placed on the use of advanced tools such as the HTS system and molecular epidemiological tools for active and wider zoonoses surveillance and monitoring, which ensures the formation of an adequate action team under a one health-based approach that includes both veterinarians and medical doctors, as well as environmental experts and other professionals.

The etiological agents may infect the host, and in advanced phases, these pathogenic agents cause worldwide epidemiology through re-transmission and amplification in varied livestock populations, as well as new zoonoses. This can result in the emergence of novel strains or species that are more virulent and/or resistant to antibiotics. Priority is given to the use of current technologies such as the HTS system and molecular epidemiological techniques (such as enzyme assay, western blot, and PCR) for active and broad zoonoses surveillance and monitoring, ensuring the formation of an adequate response team within a unified health-based framework (Fig. 2).

Bioinformatics, the area of study which focuses on methodologies for retrieving, interpreting, and archiving biological information, is an integral part of all HTS applications [39]. An NGS bioinformatic pipeline is a collection of algorithms that are run on data to produce useful interpretable results. Because proprietary NGS technological methods, commercial laboratory processes, and goals differ, the software and computational tools used by these groups will inevitably differ. Several key features are shared by all NGS bioinformatics pipelines, including sequence generation, assembly and alignment, variant identification, variant annotation, and variant prioritization and visualization. Bioinformatic pipelines also necessitate stringent quality control procedures to ensure the accuracy of the results. The metagenomics study, particularly combined with an HTS technology, has proven to be an even larger challenge for bioinformatics. Not only is the quantity of reads astonishing due to little or no filtering, but the few variables from genomics are eliminated, allowing a fluctuating number of potential genomes to map the reads against. Metagenomic analysis is thus computationally demanding, often necessitating the processing of several million reads through various sequence classification algorithms, such as the basic local alignment search tool (BLAST) for sequence similarity searches, to determine possible species in the sample. This may be experimentally avoided by targeting certain sections of the metagenome, either by sample preparation procedures or through molecular means (using amplicon-based approaches to target specific gene groups). This simplifies bioinformatics and shortens the time required for analysis.

The continued development of HTS techniques enhances the chances of detecting low copy-number pathogens by allowing deeper sequencing to be performed faster and cheaper. Importantly, sampling, sample preparation, and enrichment methods have all been shown to have a significant impact on the result of HTS-based diagnostics and thus should be considered as integral stages. Because proper diagnostic specimen sampling is critical for all investigations of animal disease. The majority of sample preparation and enrichment techniques are made up of numerous distinct processes, including homogenization, filtration, ultracentrifugation, and nuclease treatment, as well as nucleic acid extraction and purification followed by amplification. The viral genomic sequences (directly as DNA or reverse-transcribed RNA) in the samples must be translated into sequencing libraries appropriate for future cluster creation and sequencing in most existing HTS systems. The different sequencing methodologies of the major commercial HTS systems are now available. Their application areas include running time and sequence data output (Table 3).

**Table 3:** Comparison of benchtop next-generation sequencing platforms and their characteristics.

Platform	Sequencing principle	Read lengths (bp)	Throughput bases/Run	Run time	Accuracy (%)	Cost ($)/run	Instrument Cost ($)	Released date	Applications	References
Roche/454	Pyrosequencing	700 (FLX); 400 (Junior)	∼0.70 GB (FLX), 0.035 GB (Junior)	24 hrs (FLX);10 hrs (Junior)	99	∼1K (FLX); ∼6k (Junior);	∼$500 k $125 k	October (2005) (Junior); 2008 (FLX)	De novo genome sequencing and resequencing, targeted amplicon sequencing, genotyping, transcriptomics, and metagenomics	[134], [135], [136]
Illumina	Fluorescent emission from four different dyes-labeled nucleotides	36–100 (HiSeq);25–250 (MiSeq)	200–600 GB(HiSeq) ; 8.5 Gb (MiSeq)	2–5 d (HiSeq); 27 h (MiSeq)	99	∼23 k (HiSeq); ∼965 (MiSeq)	$740 k	June (2006)	Genome resequencing, targeted amplicon sequencing, genotyping, transcriptomics, and metagenomics	
NextSeq System	Fluorescent emission from two different color coding dyes-labeled nucleotides	1x75 to 2 × 150	400 million	12–30 hrs (500) 11–48 hrs (1000 & 2000 systems)	99	$1670-$6650	(N/A)	January (2014)	The NextSeq 550 System combines tried-and-true instrument technologies and tunable output with sequencing and array capabilities. The key applications are:i. Targeted sequencing (amplicon-based, gene panel) ; ii. Transcriptome sequencing (total RNA-Seq, mRNA-Seq, gene expression profiling) & Arrays	
NovaSeq 6000 (S2/S4)	It combines two-color chemistry along with patterned flow cell technology with unique dual-indexed libraries	2 × 150 to 10 billion reads	1000–3000 Gb	16–48 hrs	99	∼ $2,050 to $17,700	$850,000-$985,000	January (2017)	NovaSeq platform enables large pools of libraries to be sequenced within individual lanes of a flow cell	
ABI SOLiD	Ligation-based adapters beads and emulsion PCR	75	90 GB (SOLiD 5500); 180 GB (SOLiD 5550xI)	7 days	99.99	∼10 k	$595 k	October (2007)	Transcriptomics, genotyping, and genome resequencing	
Ion Torrent PGM	Based on the detection of hydrogen ions produced during DNA polymerization	400	2 GB	3 hrs	99	∼50 k	$350 k	2010	De novo microbial genome sequencing and resequencing, targeted amplicon sequencing, genotyping, RNA-seq on low-complexity transcriptomes, and metagenomics	
Polony sequencing	Combining end-tag library creation, template amplification, and DNA sequencing	∼26	20 GB	5–7 d	99.7	∼1k	$149 k	May 2009	De novo genome sequencing and resequencing, targeted amplicon sequencing, genotyping, transcriptomics, and metagenomics	
Pacific Biosciences SMRT	Real-time detection of single molecule DNA by fluorescence emission from the dye at the polymerase active site	1000–3000	100	1–2 hrs	85–86	∼1k	$700 k	2011	Microbial genome sequencing, as well as focused amplicon sequencing, assists in full-length transcriptomics and the detection of significant structural variations and haplotypes.	
Oxford nanopore sequencing	A nanopore and an exonuclease-based sequencing by deconstruction' method have been used to move DNA from one side of the membrane to the other.	Hundreds of Kb	Hundreds of GB	No set time (∼2–48 hrs)	90	∼0.025 to 0.040 k	$490 k	2014	Through the correct resolution of complicated genomic areas, haplotypes, and full-length transcripts, give unique and cost-effective insights on animal genomes, transcriptomes, and microbiomes Direct sequencing of native DNA or RNA also enables for the discovery of base modifications (e.g., methylation) in addition to nucleotide sequence.	

## 5 High-throughput technologies in detection of livestock pathogens

High-throughput sequencing has the potential not only to uncover novel pathogens and provide a thorough image of the virome but also to contribute to a better understanding of host responses to varied viral infections, leading to a better understanding of infection pathophysiology. The Illumina Digital Gene Expression system, which leverages Illumina deep-sequencing technology (both short and long read from HiSeq and MiSeq), was used to investigate the host response to the PRRS virus. This proved to be a strong strategy since it confirmed many of the previously known traits and expression patterns while also uncovering multiple different ones. Thus, when applied to analyses of changed gene expression in viral illness, HTS might give a better knowledge of pathophysiology, help in the development of antiviral medicines, and aid in the identification of genetic markers for resistance [40], [41], [42].

Although convenient, molecular identification methods have their limitations. False-negative findings can occur with any PCR test in case of pathogen loads below the detection limit or the presence of PCR inhibitors, affecting the utility of PCR for a broad spectrum of pathogen detection [43]. In contrast, metagenomic NGS assays have greater efficiency and allow improved infectious disease diagnostics for worldwide pathogens [44]. The advancement of genomics, which has given us access to the genomes of nearly all human pathogens, has drastically altered our approach to infectious disease management by shedding light on genetic diversity, detection, pathogenesis, evolution, and therapy [45]. NGS provides advantages over traditional microbiological diagnostic approaches in that it can detect fastidious or non-culturable organisms as well as co-infections. Next-generation sequencing platforms can generate hundreds of gigabytes of data in a single experiment, allowing for unprecedented throughput. Although the initial capital investment and cost per experiment remain high, the price per information unit (nucleotide) has been dramatically reduced in comparison to first-generation sequencing. Furthermore, these technologies enable unbiased sequencing without prior knowledge of a sample's entire DNA content, while also retaining the flexibility to allow for targeted sequencing [46].

Fetal bacteria and viruses differ widely, and the primary goal of viral identification is to link them to illness. NGS technologies are becoming an essential tool for detecting known viruses and identifying new viruses in clinical and environmental samples [47]. Viral pathogen discovery is crucial to clinical microbiology, identification of infectious diseases, and overall public health as novel viruses trigger new epidemics. In addition to infectious disease diagnosis, NGS is comprehensively used in bio forensics and biosurveillance. As a result, the widespread acceptance and applications of NGS are shifting from fundamental research to more controlled applications, including biosurveillance, bio-forensics, and clinical diagnostics [48]. Due to its high cost and lack of standardization, the whole-genome-NGS (WG-NGS) technique allows deep sequencing of nucleic acids without any a priori justification that it was initially only used as a research tool. Additionally, it is possible to directly use non-targeted identification of microbes in biological samples based on WG-NGS techniques that allow deep sequencing of nucleic acids [49].

### 5.1 Detection of pathogenic infections in bovine by sequencing technologies

Mastitis, a bovine disease caused by inflammation of the mammary gland, is the most economically significant illness in dairy cattle. Several microbes have been identified as the source of this disease, and managing mastitis is becoming difficult [50]. It is a lethal mammary gland infection that leads to a massive loss in the dairy industry. Disease-causing bacteria are often referred to as infectious agents. These bacteria can be classified as environmental or contagious depending on the mode and source of transmission. The most regular mastitis pathogens are classified as contagious pathogens (*Staphylococcus aureus,* etc.) or environmental pathogens like animal dung, soil, etc.

Environmental pathogens are now widely recognized as the primary cause of clinical mastitis. To define clinical mastitis in cows on large dairy herds in Wisconsin, 50 herds were evaluated. Microbiological studies have been conducted to describe mastitis pathogens and have revealed that gram-negative bacteria are the most common cause of clinical mastitis [51]. Recently, bacterial DNA diversity in milk samples from mastitic and healthy dairy cows was also investigated using metagenomics of bacterial 16S rRNA genes [52].

Our understanding of bovine respiratory health is constantly evolving in response to technological developments. Many techniques, such as serology, microbial culture, and PCR, have been utilized for pathogen detection in cattle. Today, NGS appears promising, and due to the democratization of technology, NGS technologies (HiSeq and MiSeq) are now becoming more widely available. NGS is beneficial in that all pathogens may be discovered simultaneously, without having to prioritize the detection of a pathogen [53]. Bovine respiratory disease (BRD) is caused by pathogenic bacteria in lung tissue and is associated with significant morbidity and mortality in cattle worldwide.

As discovered by NGS, *Pasteurellaceae, Leptotrichiaceae, Mycoplasma*, and *Fusobacterium* are the most abundant bacteria in the lungs and lymph nodes of BRD-dead calves [54]. BRD has also been detected using metagenomic sequencing to describe the respiratory viromes of paired nasal swabs and tracheal washes in Canadian cattle causing economic losses in North America. The findings revealed that influenza D virus, bovine rhinitis B virus, and bovine rhinitis A virus are the most abundant viruses found in nasal swabs in BRD, whereas the bovine respiratory syncytial virus is primarily found in tracheal washes and bovine coronavirus in both the nasal and tracheal areas [55].

### 5.2 Sequencing technologies and pathogens in detection *canis lupus familiaris*

Canine vector-borne diseases are a collection of infectious disorders spread by arthropod vectors, such as ticks and other pathogens, such as bacteria and protozoa [56]. Ticks and related infections in dogs in northern Vietnam have been characterized at the molecular level [57]. In young dogs, canine parvoviral enteritis is a severe cause of morbidity and mortality. Parvoviruses are small, non-enveloped, single-stranded DNA viruses that cause illnesses in several mammalian species. CPV spreads quickly in dogs by fecal-oral transmission (direct transmission) or oronasal exposure to feces-contaminated fomites. Full-length genomes and deep sequencing have been performed on 40 puppy feces samples to assess the genetic divergence between the CPV-2a and CPV-2c variants and to investigate the incidence of co-infection and recombination events [58].

Dog bites can cause severe tissue damage as well as the transmission of bacterial infections and rabies. Rabies is a well-known zoonotic illness that has been around for approximately 4,300 years. It is a global zoonosis caused by a lyssavirus, with several host species, primarily dogs, acting as reservoirs for infection [59]. The rabies virus (RABV) causes an acute, deadly brain infection in humans and other species. It is transmitted through a bite or scratch from a rabid animal's saliva [60]. Researchers were able to compare the deep genetic diversity and evolution of RABV subpopulations, microevolution, and adaptability patterns success can be attributed to the Illumina MiSeq platform [61]. Bacterial vector-borne diseases, such as Rickettsia bacteria in the tropics, *Anaplasma platys,* and *Ehrlichia canis*, commonly cause canine cyclic thrombocytopenia and canine monocytic ehrlichiosis [62].

Vector-borne pathogens *Anaplasma platys, Babesia gibsoni, Babesia vogeli, Ehrlichia canis, Hepatozoon canis,* and *hemotropic Mycoplasma* spp. are all endemic in the Asia-Pacific region and can be detected using qPCR. The combination of qPCR with NGS technology provides a valuable high-throughput diagnostic tool for epidemiologists, researchers, and clinicians [63]. Bocaviruses are small, non-enveloped DNA viruses that belong to the Parvoviridae family's *Bocaparvovirus* genus and have been associated with respiratory and gastrointestinal diseases in both humans and animals. CBoV-2 has also been discovered in NGS data of lung samples and confirmed through traditional PCR experiments [64].

Haemoparasites cause some of the most common and debilitating diseases in dogs in the world and pose a considerable zoonotic threat to humans. The sequence-metabarcoding-based technique has been used to test a variety of blood-borne apicomplexan and kinetoplastid parasites in Bangkok and Thailand's temple dogs, demonstrating how NGS techniques can cover atypical infections [65].

### 5.3 Detection and identification of various agents involved in *Ovis aries* infectious

Pneumonia and scours are the leading causes of death in lambs. *Pasteurella* spp. is the most common etiological agent linked with pneumonia in lambs, while *Escherichia coli* is in non-parasitic scours. Pneumonia is also the most common cause of death in ewes, and although *Pasteurella* spp. appear to be the most common etiological agents, agents vary with location [66]. Ticks are implicated in the spread of various viruses that have serious health consequences in sheep. Several unique and divergent tickborne viruses have been documented to exist and circulate worldwide. Ticks have also been associated with the spread of various other infectious diseases with significant human and animal health implications [67].

Internal parasites, sheep scabs, and footrots have been identified as significant diseases in sheep by the Moredun Foundation (1997). Lambs are constantly challenged by gastrointestinal parasites, which are only temporarily inhibited by anthelmintic treatment. Gastrointestinal nematodes are one of the most common parasites found in domestic sheep worldwide. To detect CNV associated with disease, researchers have used data from NGS. CNV is a variation that contributes to genetic diversity and disease features. However, CNVs in sheep are understudied compared to those in other domestic animals [68].

Along with G.I. nematodes, multiple co-infecting trichostrongylid nematode species, each with a different incidence of benzimidazole resistance, make sheep an excellent host system. Deep amplicon sequencing has been developed and validated as a powerful method for detecting and quantifying the frequency of SNPs linked to benzimidazole resistance. It is a new approach for identifying anthelmintic resistance mutations in parasitic worm populations in both animals and humans [69]. Footrot is common in most sheep-producing countries, where it causes significant economic losses and jeopardizes the health and welfare of sheep. Swabs from the hooves of Merino sheep from New South Wales, Australia analyzed 16S rRNA revealing the presence of different bacterial populations on the feet of healthy and footrot-affected sheep. Gram-positive, aerobic taxa were predominant on the feet of healthy sheep, whereas gram-negative and anaerobic taxa were predominant on the feet of footrot-affected sheep [70].

*Mycoplasma agalactiae* is a dangerous pathogen of small ruminants that spreads mainly through the mammary system, causing acute to subacute mastitis that progresses to a chronic, difficult-to-cure illness. Mycoplasma agalactiae is a species of bacteria in the genus Mycoplasma are the smallest bacterial cells so far discovered that lack a cell wall around their cell membrane [71]. Because of this reason, antibiotics that target cell wall synthesis are unaffected. Mycoplasma agalactiae can cause mastitis in the animal and make serious outbreaks of infections with this pathogen. Using Illumina RNA-sequencing, researchers compared the transcriptome profiles of mammary tissues from sheep experimentally infected with *M. agalactiae* type strain PG2. It was the first study to look at the host transcriptomics of *M. agalactiae* infection and the corresponding immune-inflammatory responses [72]. Recently, miRNAs have been identified as novel targets for revealing disease molecular processes because of their variable expression through diseased and healthy states. Low-abundance proteins, such as cytokines, have been quantified using ELISA, qRT-PCR, and proteomic techniques as well as qRT-PCR and NGS [73].

### 5.4 Sequencing technologies for detection of pathogenic infections in pigs

*Klebsiella pneumoniae* is a common harmless bacterium of the intestines, but its migration into the other parts of the body turns it into a superbug that is practically impossible to fight with widespread antibiotics [74]. ABI SOLiD sequencing platform facilitated the detection of antibiotic resistance in *Klebsiella pneumoniae* strains isolated from hospitals in Israel [75]. Recently, the sequences colistin-resistant *K*. *pneumoniae* strains were isolated from swine farm in Malaysia [76] and the sequences of three multi-resistant plasmids in a *K*. *pneumoniae* isolate from swine in China have been characterized [77]. PacBio sequencing, developed by Pacific Biosciences, captures sequence information by single-molecule real-time sequencing. PacBio RSll sequencing involves the addition of nucleotides labeled with a dye to a template DNA strand by DNA polymerase and fluorescence signal detection in real-time.

The PacBio method is suited for de novo assembly and base modification detection [78]. The disadvantage of the PacBio method is the cost of the instrument and the amount of DNA template required. However, the PacBio technique is the most accurate and complete genome production method [78]. The PacBio platform was used to detect antimicrobial resistance genes in *Erysipelothrix* rhusiopathiae, a strain isolated from swine, through the identification of novel chimeric integrative and conjugative elements responsible for drug resistance [79]. PacBio platform was also used to investigate the antibiotic resistance genes in novel plasmid variants (pAsa5-3432 and pRAS3-3432) in *Aeromonas salmonicida* aquatic pathogen and establish a link with swine pathogen to transfer antibiotic resistance gene [80]. In addition, multidrug-resistance in *E. coli,* as well as resistance genes carrying two novels Proteus genomic island 2 (PGI2) variants in *Proteus mirabilis* were studied using the PacBio platform [81]. In 2014, Oxford Nanopore released a portable nanopore device wherein a single DNA or RNA molecule can be sequenced without PCR amplification or chemical labeling using the nanopore technique. A single band of a DNA molecule is guided through a protein nanopore, which develops an electrical current across a lipid membrane [82]. Nanopore has a variety of applications in many areas, including DNA, RNA, and protein sequencing and drug development. A MinION sequencer generates a maximum read length of more than 50 kilo bases [83]. The Oxford Nanopore platform has exceptional potential for use as a rapid disease diagnostic tool due to its high mobility, accessibility, and short turnaround time [82]. This platform was used to analyze the antimicrobial resistance profile of *Streptococcus suis* [84]. Moreover, the *Tet(63)* novel tetracycline resistance gene from *Staphylococcus aureus* was identified through the genome sequencing platforms Illumina HiSeq and Oxford Nanopore [85].

### 5.5 Detection of pathogenic infections in chicken and duck through NGS platforms

Influenza A virus sequencing costs were reduced by 92 % from 2010 to 2015 due to new technologies [86]. Because of the NGS approach, the influenza virus variant PIV4 subtype was successfully identified in late 2013 [87]. The NGS platform Roche 454 GS FLX System was used to analyze zoonotic influenza in a virus-infected lung tissue sample from the ferret in 2009 [88]. In addition, the eight known strains of West Nile virus were sequenced using the 454 sequencing platform [89]. Ion Torrent technology creates a direct connection between chemical changes due to nucleotide binding and signal detection. Ion Torrent is similar to pyrosequencing technology; however, it differs from other techniques, such as the Ion Torrent personal genome machine and Ion Proton sequencer, which detect H^+^ release on ion semiconductor sequencing chips during the binding of new nucleotide in the strand by DNA polymerase and generates read lengths of around 200 bp lies in between short and long read length NGS technologies [82], [90]. The method is most suitable for small genome sequencing and targeted sequencing methods [82]. RNA-seq, or sequencing-based transcriptomics, has been used to study the transcriptome response in chicken spleen to avian pathogenic *E. coli* (APEC) infection, as well as the integrated expression of miRNAs and mRNAs in the lungs of AIV-infected broilers. In the APEC investigation, potential genes for host response were discovered inside critical physiological pathways such as the *T*-cell receptor (TCR) signalling pathway.

The whole-genome sequencing of the most common foodborne disease-causing bacteria, such as *Campylobacter jejuni*, *Listeria monocytogenes*, and *Salmonella enterica,* via different NGS platforms, has suggested that integrated data analysis based on Illumina and Ion Torrent is a more suitable method [91]. The Ion Torrent platform was used to sequence the highly pathogenic H5N2 and H7N1 avian influenza virus genomes. Mutations in both strains and their pathogenic potential were identified, with highly pathogenic viruses emerging from less pathogenic strains [92].

### 5.6 Detection of pathogenic infections in horse by NGS platform

Illumina Genome Analyzer IIx was used to analyze the viral heterogeneity of rabies genomes in infected tissues, which allowed for extensive and comprehensive genome sequencing of the EEE virus [93]. Methicillin-resistant Staphylococcus aureus (MRSA) bacteria resistance against several antibiotics facilitates infection in the horse and can be found in skin wounds and various sites in the respiratory tract. The MRSA was isolated and whole-genome sequenced (WGS) using Illumina MiSeq 300 bp paired-end sequencing [94]. It spreads through skin-to-skin contact; therefore, it poses challenges to normal animals, clinicians, and farm employees. The knowledge acquired from utilizing RNA-seq to gain an overview of pathogen-induced host responses or to track temporal expression changes over the infectious cycle can be used to create better control measures and to find genetic markers for resistance.

## 6 Outbreak management and zoonotic transmission control

**Fig. 3:**
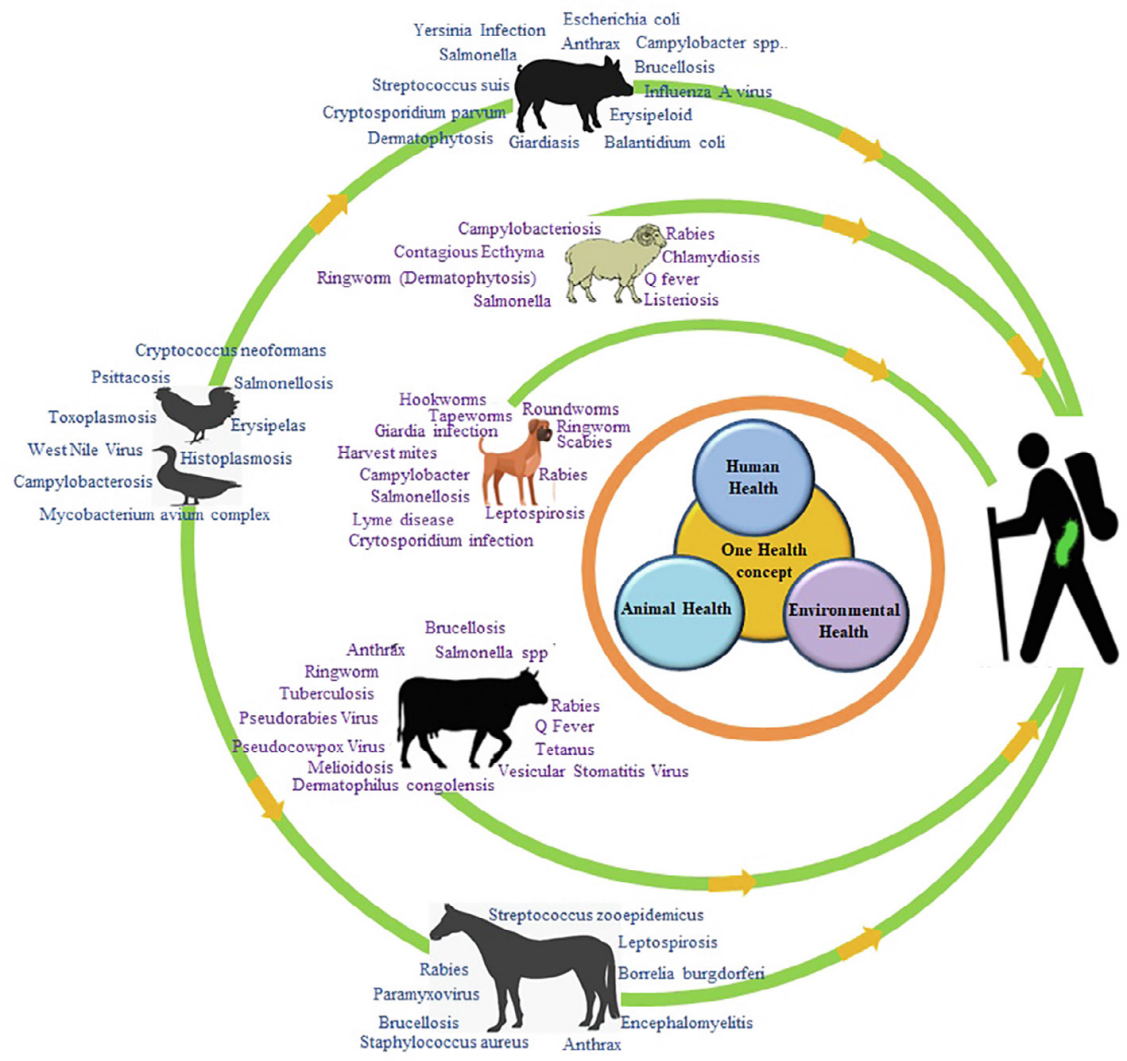
Overview of impact of disease transmission from cows (*Bos taurus*), pigs (*Sus scrofa*), sheep (*Ovis aries*), goats (*Capra hircus*), chickens (*Gallus gallus domesticus*), and ducks (*Anatidae*)–the etiological agents of emerging and re-emerging zoonoses. The presence of agents in atypical hosts can often increase the risk of abnormalities in the process of RNA replication, which can lead to mutations. This can result in the emergence of novel strains or species that are more virulent and/or resistant to drugs. Increased transmission rates in sensitive groups might be one of the consequences.

Pathogen infections in wild and domestic animals pose a global health risk concerning sporadic human zoonotic infections. Recognized zoonoses have increased to over 200 due to ecological, climatic, and sociocultural changes. Zoonoses can be categorized into emerging, reemerging, and neglected classes of diseases [95], [96]. Emerging and reemerging diseases have significant impacts on public health and socio-economic status globally. Targeted disease surveillance, data analysis, and database search for all reported emerging infectious diseases to analyze the major transmission pathways could facilitate the optimization of prevention and control measures, and minimize future zoonotic disease threats. Several zoonotic vector-borne diseases have possible human health consequences (Fig. 3) and therefore, call for management [97]. High-impact epidemics have occurred in the past decades due to pathogenic invasions from wild animals and livestock reservoirs (Table 4). Therefore, the risk of zoonoses emerging from animal populations should not be overlooked.

**Table 4:** Zoonoses emerging from domestic animals and posing risks of sporadic human zoonotic infections.

Animal	Disease	Causative agents	Description	Ref
Bovine	Anthrax	*Bacillus anthracis*	*Bacillus anthracis*, a large rectangular spore-producing bacterium causes anthrax, a highly infectious and fatal cow disease. Although drugs against anthrax are clinically used, treatment is often unavailable due to the sensitive nature of the condition, leading to quick mortality.	[137]
	Black quarter	*Clostridium chauvoei*	It is a highly contagious and deadly bacterial disorder in cattle caused by *Clostridium chauvoei*. Buffaloes, sheep, and goats are among the animals that are impacted. Early therapy may lead to total recovery.	[138]
	Bovine spongiform encephalopathy	BSE prion	Bovine spongiform encephalopathy (BSE) is a neurodegenerative disease in cattle caused by prions. It affects the central nervous system of adult cattle. The first case of BSE, also known as “mad cow disease,” was discovered in the United Kingdom in April 1985.	[139]
	Bovine tuberculosis	*Mycobacterium bovis*	Bovine tuberculosis (TB) caused by *Mycobacterium bovis* leads to the formation of granulomatous lesions or tubercles over time in the lung tissue, lymph nodes, or other organs. TB is contagious among bovine species, such as bison and buffaloes, but it may infect nearly all warm-blooded animals.	[140]
	Bovine diarrhea	Bovine viral diarrhea virus	Bovine viral diarrhea (BVD) is caused by the bovine viral diarrhea virus (BVDV). The symptom of BVD varies depending on the immune status of the exposed animals and the strain of the infecting virus. BVD presents as an acute severe sickness with bloody diarrhea, high fever, off-feed, mouth ulcers, and pneumonia.	[141]
Dog	Rabies	Rabies virus	Rabies is one of the oldest identified human diseases and one of the most important zoonotic diseases in India. It affects both aerial and terrestrial animals, such as dogs and wolves. Clinical symptoms and indicators as well as a corroborative narrative of or proof of an animal bite are used to diagnose human rabies.	[142]
	Lyme disease	Borrelia burgdorferi	Lyme disease is a tickborne, systemic infection with a wide range of symptoms. The development of erythema chronicum migrans, a pathognomonic skin lesion, generally precedes the beginning of the disease. Usually, clinical evidence and serologic test findings are used to define Lyme disease.	[143]
	Ehrlichiosis	*Ehrlichiosis canis*	Tickborne diseases are becoming an increasingly severe public health issue. Multiple outbreaks of novel tick-borne diseases and subsequent identification of their origins have raised public awareness about tickborne diseases.	[144]
	Gastrointestinal infection	*Canine parvovirus*	Highly infectious viral dog disease causes acute Global Developmental Delay (GDD) in puppies. The most common disease occurs in 6- to 20-week-old puppies; however, older animals are sometimes afflicted. In young puppies, myocarditis is an uncommon form of the disease.	[145]
Sheep	Sheeppox, Goatpox	Sheeppox virus, Goatpox virus	The capripoxvirus causes sheeppox and goatpox. The condition is less common in indigenous than in alien breeds. The infection is mostly spread through direct contact.	[146]
	Contagious caprine pleuropneumonia	Contagious caprine pleuropneumonia	Pleuropneumonia is a deadly and contagious infection in goats. It is transferred by infective aerosol. Clinical symptoms, epidemiology, and necropsy results are used for diagnosis.	[147]
	Salmonellosis	*Salmonella* spp.	Salmonellosis is a bacterial infection that causes sickness and mortality in sheep. *Salmonella* multiplies in the gastrointestinal system and other organs, causing infection. It presents with diarrhea and may lead to abortion in sheep.	[148]
	Scrapie	*Scrapie prion*	Scrapie is a deadly neurodegenerative disease in sheep and goats. It is caused by an aberrant prion, a deviant version of a benign protein that typically presents in the brain.	[149]
Pigs	Influenza	Influenza A virus	Influenza A viruses belonging to the family Orthomyxoviridae cause acute respiratory infections. They have a genomic single-stranded RNA with polymerase lacking proofreading ability. Therefore, influenza A viruses have a high mutation rate. Three different subtypes of influenza A viruses of swine are known (H1N1, H3N2, and H1N2). Influenza A virus targets the epithelial cells of the respiratory tract and replicates primarily in the lungs. Symptoms of influenza include fever, respiratory distress, and weakness and may lead to death. It can also lead to weight loss in growing pigs and subsequent economic loss.	[150]
	Giardiasis	*Giardia lamblia*	Giardiasis leads to bouts of diarrhea, especially in young animals, which adversely affects production, resulting in economic losses. It is caused by *Giardia lamblia*, one of the most widespread intestinal protozoan parasites in humans and other animal species worldwide. The disease spreads by the fecal-oral route, either by direct contact with an infected host or through contaminated food or water.	[151]
	Yersinia infection	Yersinia pseudotuberculosis, *Yersinia enterocolitica*	*Yersinia* is the third most frequently reported zoonotic disease-causing foodborne intestinal illness in humans. It is transferred to humans by food contaminated with animal feces. The symptoms may include fever, abdominal pain, diarrhea, nausea, and vomiting.	[152]
	Dermatophytosis	*Microsporum canis*	Dermatophytosis is infectious to humans and many species of animals, including pigs. It occurs in any part of the body, mainly in the back, thorax, and flanks.	[153]
	Psittacosis	*Chlamydia psittaci*	Psittacosis affects many birds and some mammals. It is transmitted from bird to bird or bird to mammal through food, water, or dust particles. It can cause diarrhea, respiratory issues, and weight loss.	[154]
Chicken and duck	Toxoplasmosis	*Toxoplasma gondii*	Toxoplasmosis is a protozoan parasite infection in humans and animals. Birds can get infected with toxoplasmosis through the ingestion of the parasite’s oocysts or the tissue of infected animals. Thus, the infection may be transferred through the food chain from wild to domestic animals. The symptoms of the disease include neurological, ocular, and pulmonary issues or multi-organ infection.	[155]
	Histoplasmosis	*Histoplasma capsulatum*	*Histoplasmosis* is caused by inhaling the spores of the fungus *Histoplasma capsulatum* and can spread through air or contaminated soil to reach the host lungs. It can cause tuberculosis- or influenza-like symptoms and turn fatal, if untreated.	[156]
	Cryptococcus	*Cryptococcus neoformans*	*Cryptococcus* causes acute or chronic lung infection in mammals, including humans. Pathogen virulence depends on the thickness of the *Cryptococus neoformans* capsule. The infection transfers through bird droppings wherein fungus enters the host via dust inhalation and through the skin.	[157]
	Hendra virus disease	Hendra virus	*Hendra virus*, a member of paramyxovirus, causes acute and deadly infection in horses and spreads from fruit bat to horse or horse to horse. Hendra virus disease causes substantial economic loss to the sports horse industry. It first emerged in 1994 in Australia. It presents with fever, increased heart rate, respiratory distress, weight shifting between legs, and apparent vision loss. Transmission occurs through urine, saliva, fluids, or bats.	[158]
	Lyme disease	Borrelia burgdorferi	Lyme disease is transferred to animals and humans through tick bites. *Borrelia burgdorferi* is not a free‐living organism, and it maintains a zoonotic life cycle in mammals or ticks. The clinical signs of the disease include neuroborreliosis, uveitis, lameness, and stiffness.	[159]
Horse	Rabies	Rabies virus	Described at the dog pathogens section.	
	Anthrax	*Bacillus anthracis*	See above under bovine pathogens	

### 6.1 Monitoring and detection of zoonotic infections by HTS

The Global Health Security Agenda (GHSA) was launched in 2014 to monitor and help prevent zoonotic diseases for global health security [98]. According to the pathogen discovery approach, the program implements zoonotic disease detection, prevention, and control initiatives, to generate knowledge that could facilitate the early discovery of emerging zoonotic pathogens or reemerging diseases. Metagenomic NGS has been used to detect rare and new viral etiologies and describe viral diversity in human, animal, and environmental samples [99]. Successful metagenomic next-generation sequencing (mNGS) can be achieved using rapidly developing NGS technologies. Recent zoonotic outbreaks worldwide were quickly controlled by high-throughput technologies and provided new perspectives on the zoonotic transmission of microorganisms [100].

Buffalopox or pox-like illnesses have been observed in buffaloes, cows, and humans in many parts of the world. Since its initial outbreak in India, many epidemics have followed. Buffalopox virus, the primary agent of the disease, belongs to the genus Orthopoxvirus. Disease control is challenging in nations where the disease is prevalent, and animal migration is difficult to manage [101]. In 2016, another outbreak was reported in the interiors of Alaska following which, rectal-swab specimens from dogs with clinical symptoms compatible with parvoviral-associated diseases were analyzed using sequencing techniques. The target RNA transcripts identified CPV-2a and CPV-2b strains, which helped manage the outbreak [102].

*Mycobacterium chimaera (M. chimaera*), a nontuberculous mycobacterium that belongs to the *Mycobacterium avium* complex, is an opportunistic and ubiquitous human pathogen found especially in water sources. Patients exposed to contaminated heater-cooler equipment used during heart surgery contracted *M. chimaera* infections. *M. chimaera* infection can be diagnosed using a plasma-based NGS test [42]. NGS is useful in the management of outbreaks. On November 24, 2015, the French National Reference Laboratory for avian influenza reported a highly pathogenic H5N1 avian influenza epidemic in backyard layers and hens in the Dordogne Department, South-Western France. To discover and characterize this epidemic virus, full genome sequences were generated using NGS, and the outbreak was successfully controlled [103]. Despite its many advantages, there are several limitations to the whole-genome sequencing of epidemic pathogens. One of them is the inability of NGS to discriminate between living and dead pathogens. The requirement for simplifying samples by selective culture before sequencing, up-to-date large-scale informatics databases, and the development of reference databases are constraints of existing whole-genome sequencing methods [104]. The coronavirus disease 2019 (COVID-19) epidemic started on December 12, 2019, in Wuhan City, China [105], and spread globally with over 224 million cumulative cases and over 4.6 million deaths [106]. Scientists hold contentious ideas about COVID-19 as a zoonotic disease or an emerging infectious disease [107].

Whole-genome sequence analysis of the SARS-CoV-2 virus revealed that it shares an identity with a horseshoe bat coronavirus (96.2 %) and SARS-related coronaviruses (79.6 %) [108]. Coronavirus is a single standard positive sense RNA enveloped virus and includes the genera alpha, beta, gamma, and delta of the Coronaviridae family [109]. According to phylogenetic studies, bat pathogens predominantly serve the alpha and beta forms [110]. The SARS-CoV-2 virus can use the entry receptor angiotensin-converting enzyme II (ACE2). The infection by SARS-CoV-2 is much more contagious due to its high-affinity binding capability with the ACE2 receptor [111]. Six strains of coronaviruses were identified to infect humans and three zoonotic coronaviruses (SARS-CoV, MERS-CoV, SARS-CoV-2) produce serious symptoms and cause severe disease in livestock and companion animals such as pigs, cows, chickens, dogs [112]. Sequence analysis indicated that the primary host was bat, however, the intermediate host is yet to be discovered. Based on NGS results, scientists warned about the new zoonotic virus before identification of the virus [113]. Ultimately, NGS offers great promise in enabling scientists to trace novel and emerging zoonotic diseases and prevent the loss of millions of human lives or economic catastrophe.

### 6.2 Vaccine development

Because of its inherent benefits as a screening and characterization technique, HTS is suited for application in poultry vaccine innovation and quality control [114], [115]. A genome-level comparison of two United States lives attenuated infectious laryngotracheitis virus (ILTV) vaccines with an Australian strain demonstrated that high-throughput sequencing may be utilized to distinguish among vaccination strains [116], [117], [118].

An intriguing use is the investigation of interactions between emerging field strains and vaccination strains. Recent research on infectious ILTV showed that separate recombination processes between different attenuated vaccination strains caused virulent recombinant viruses which became the dominant strains responsible for widespread illness in commercial Australian chicken flocks [119]. This shows the consequences of combining numerous attenuated vaccinations or vectors in the same populations.

Using HTS, the researcher can now obtain an insight into the mechanisms that result in attenuating mutations throughout the serial passage of a virulent strain. Although the above examples are for bird vaccines, it should be noted that comparable HTS procedures may be used for vaccines used in many other animal species, even humans.

## 7 Conclusion and perspectives

NGS technologies have completely transformed the field of genomics. Nevertheless, there are several significant limitations to HTS-based pathogen inferences. In environmental samples, the metagenomic process tends to compound errors, obscuring inferences about pathogen diversity. Because pathogens and parasites are so diverse, developing primers to target numerous pathogens for metabarcoding is difficult. Furthermore, pathogen database coverage is significantly lower than in other organisms, resulting in identification uncertainty and inaccurate estimates of pathogen richness. Nevertheless, costs are still high, particularly in areas with the greatest diversity of pathogens and those most likely to have an adverse effect on both human and animal health. Determining pathogen virulence in humans or their domestic animals is also a crucial challenge. Although it is still difficult to predict when an outbreak of infectious disease will occur, HTS methods have significant benefits for enhancing urgently required surveillance and expanding our knowledge of infectious diseases.

## Funding

This work was supported by the National Research Foundation of Korea grant funded by the Korean government (2020R1A2C2004128) and the Basic Science Research Program through the National Research Foundation of Korea funded by the Ministry of Education (2019R1A6A1A10072987). The authors are thankful to the Brain Pool Program supported by the Ministry of Science and ICT through the National Research Foundation of Korea (2022H1D3A2A02053110).

## CRediT authorship contribution statement

**Godagama Gamaarachchige Dinesh Suminda:** Conceptualization, Data curation, Writing – original draft. **Srishti Bhandari:** Conceptualization, Data curation, Writing – original draft. **Yoonkyung Won:** Resources, Data curation. **Umesh Goutam:** Writing – review & editing. **Krishna Kanth Pulicherla:** Formal analysis, Investigation, Validation. **Young-Ok Son:** Formal analysis, Investigation, Supervision, Writing – review & editing. **Mrinmoy Ghosh:** Conceptualization, Writing – review & editing.

## Declaration of Competing Interest

The authors declare that they have no known competing financial interests or personal relationships that could have appeared to influence the work reported in this paper.
